# Adenosine A2a Receptor Regulates Autophagy Flux and Apoptosis to Alleviate Ischemia-Reperfusion Injury *via* the cAMP/PKA Signaling Pathway

**DOI:** 10.3389/fcvm.2022.755619

**Published:** 2022-04-29

**Authors:** Yun Xia, Feng He, Mohamed Bassirou Moukeila Yacouba, Huimin Zhou, Jingfan Li, Ying Xiong, Jingjing Zhang, Hui Li, Yanlin Wang, Jianjuan Ke

**Affiliations:** Department of Anesthesiology, Zhongnan Hospital of Wuhan University, Wuhan, China

**Keywords:** adenosine A2A receptor, autophagy, autophagosomes, myocardial ischemia-reperfusion injury, apoptosis

## Abstract

Exploring effective methods to lessen myocardial ischemia-reperfusion injury still has positive significance. The adenosine A2a receptor (A2aR) has played a crucial part in cardiac ischemia-reperfusion injury. Previous studies revealed that the adenosine A2a receptor regulated autophagy, but the specific mechanism in myocardial ischemia-reperfusion injury was still unclear. We established an ischemia-reperfusion model (30 min of ischemia and 2 h of reperfusion) *in vivo* and a model with oxygen-glucose deprivation for 6 h and reoxygenation for 18 h (OGDR) *in vitro*. The ischemia-reperfusion injury resulted in prolonged QTc interval, left ventricular systolic dysfunction, and myocardial infarction. *In vitro* model, we found that the OGDR-induced autophagosomes and apoptosis caused myocardial cell death, as evidenced by a significant increase in the generation of lactate dehydrogenase and creatine kinase-MB. Furthermore, overactivated autophagy with rapamycin showed an anti-apoptotic effect. The interaction between autophagy and apoptosis in myocardial ischemia-reperfusion injury was complex and variable. We discovered that the activation of adenosine A2a receptor could promote the expression of Bcl-2 to inhibit the levels of Beclin-1 and LC3II. The number of autophagosomes exceeded that of autolysosomes under OGDR, but the result reversed after A2aR activation. Activated A2aR with its agonist CGS21680 before reperfusion saved cellular survival through anti-apoptosis and anti-autophagy effect, thus improving ventricular contraction disorders, and visibly reducing myocardial infarction size. The myocardial protection of adenosine A2a receptor after ischemia may involve the cAMP-PKA signaling pathway and the interaction of Bcl-2-Beclin-1.

## Introduction

The pathological basis of coronary heart disease (CHD) is myocardial ischemia, hypoxia, and necrosis caused by coronary artery occlusion and stenosis. It remains higher global morbidity and mortality. For acute myocardial infarction (AMI), as a kind of CHD, reperfusion and revascularization are the conventional treatments used in clinical. However, vascular reperfusion will inevitably lead to myocardial ischemia-reperfusion injury (MIRI) complications involving a sequence of pathophysiological and metabolic alterations. Severe MIRI will result in myocardial failure, arrhythmia, and death of myocardial cells, mainly caused by hypoxia, changes in cytoplasmic pH, calcium overload, ATP deficiency, and immune cell aggregation (1). The occurrence of MIRI will change the disease outcome and treatment of some patients with AMI (2). Therefore, some approaches are needed to reduce the incidence of MIRI or its extent of damage.

Adenosine receptor (AR), a type of glycoprotein, exists on the membrane of most cells of the body. It has a critical effect on the cardiovascular system by cascading with numerous effectors such as enzymes, channels, transporters, and cytoskeleton (3). Multiple angiocardiopathy, for instance, hypertension, atherosclerosis, diabetic cardiomyopathy, and ischemic heart disease have been related to changes in adenosine-adenosine receptor signaling in coronary microcirculation (4). There are three types of AR: A1R, A2R, and A3R, of which A2R has two subtypes, A2aR and A2bR. Adenosine A2a receptor (A2aR) most widely distributes in coronary vessels (5). It exerts a positive significance in adjusting MIRI. Previous studies showed that A2aR's activation could alleviate MIRI in normal hearts (6, 7) or sepsis ones (8), in stunned myocardium (1), and reduce mitochondrial oxidative stress after reperfusion (9). The cardioprotective effects of A2aR in MIRI also include the inhibition of the inflammatory response (10) [CD4^+^ T lymphocytes (11) and mast cells (12)] and apoptotic cell death (13). Moreover, the combination of activators with A2aR can activate adenosine cyclase (AC) and increase the formation of cyclic adenosine monophosphate (cAMP) (14). As a second messenger, cAMP promotes the generation of protein kinase A (PKA), which makes downstream proteins phosphorylation to form the cAMP-PKA signaling pathway (15). The cAMP-PKA signaling pathway also relates to inhibition of myocardial fibrosis (16) and limitation of MIRI (17).

Autophagy is an important mechanism for preserving the dynamic balance of the intracellular environment (18–20). It undertakes the degradation and recycling of intracellular organelles and proteins, as well as the secretion and transportation of intracellular substances (21). Current researches have shown that autophagy appears to play markedly different roles during ischemia and reperfusion period. It is unanimously considered that autophagy in ischemia is pivotal to maintain the stability of cardiac function and lessen myocardial damage. During the ischemic period, autophagy, activated in response to a lack of oxygen and nutrients, can promptly resolve harmful substances and damaged organelles (22). The startup of this self-preservation program not only reduces the cardiomyocytes' damage but also provides amino acids, fatty acids, ATP regenerative substrates as supplements to protein synthesis and ATP generation for cell survival (19, 22). However, the role of autophagy in reperfusion seems to be a debate (23, 24). Unlike the effect of energy recovery, over-activated autophagy may cause cell death and further increase the damage. Thus, that is considered harmful. In the case of using Atg7 knockout mice to impair autophagy function, the production of ROS decreased, and the activity of myocardial cells enhanced after reperfusion (25). But contrary views (26, 27) claimed that the increase of autophagy during reperfusion had a previously undescribed and formidable protective response on MIRI. In addition, autophagy in MIRI has a complex relationship with apoptosis, and their interplay and balance also provide a new treatment concept for coronary heart disease (24).

Our earlier research (7) suggests that A2R activation can suppress autophagy during reperfusion, which dues to the A2bR subtype. A2aR activation alone, however, seemed cannot decrease the infarcted area of heart. Considering the beneficial expression of A2aR in other reports, we reconsider whether its potential is underestimated in autophagy of MIRI. Due to the limited research on A2aR regulating autophagy in MIRI, we designed this experiment.

## Materials and Methods

Detailed information about related compounds, reagents, antibodies were provided in the attached table (Supplementary Tables 1, 2).

### Animals

All Sprague Dawley adult rats (250 g−280 g) and neonates (1–3 days) were offered by Hubei Experimental Animal Research Center. Animal management and experimental protocols following the Guide for the Care and Use of Laboratory Animals (National Institutes of Health) were approved by the Animal Experimentation Committee of Wuhan University (approval number WP2020-01108).

### Extraction and Purification of Myocardial Cells

Neonatal rat cardiomyocytes (NRCMs) were extracted from the neonates of 1–3 days, according to the improved method based on the classic protocol (28). Ventricles were separated from large vessels, atria, and pericardium in a 10 cm petri dish with DMEM/F12 medium at 4°C, and subsequently transferred into a 20 ml beaker, containing the digestive juices of PBS, 0.125% trypsin, and 0.08% type II collagenase for tissue digestion. We added a 1 cm stir bar in beaker and put the beaker on a magnetic blender, which was set at 220 rotations per min (rpm) to facilitate the digestion. And the beaker and magnetic blender were all put in the digital biochemical incubator at 37°C. Each digestion lasted 10 min, with a total of 12-14 times. After each digestion, the digestive juice was transferred to a 15 ml test tube to terminate digestion with DMEM/F12 supplemented with 10% fetal bovine serum (FBS), 1% penicillin-streptomycin and 1:1000 5-Bromo-2′-deoxyuridine (BrdU). Next, the cell deposition was re-suspended with the fresh medium after 5 min centrifugation at 4000 rpm. Then the cell suspension was filtrated with a sterile 70 um filter into the 10 cm cell culture dishes for 1 h incubation to remove the adherent fibroblasts. Eventually, the cell suspension was filtrated again and 2× 10∧6 cells was planted into each 6 cm dish after cell counting (Countstar^®^ BioMed, Shanghai). In addition, immunofluorescence identification was performed to evaluate the purification of cardiomyocytes.

### Establishment of OGDR Model *in vitro*

NRCMs cultured *in vitro* were treated with oxygen-glucose deprivation and reoxygenation (OGDR) to simulate ischemia-reperfusion injury *in vivo*. Cardiomyocytes were cultivated in a glucose-free, serum-free medium with 95% N_2_ and 5% CO_2_ for 6 h. Then, the fresh DMEM/F12 supplemented with 10% FBS, 1% penicillin-streptomycin, and 1‰ BrdU was used again and reoxygenated for 18 h incubating at 37°C and 5% CO_2_ (29).

### Cardiomyocytes' Identification

When myocardial cells were grown for 24 h, immunofluorescence was performed with cardiac troponin I (TNNI3) (30) as the primary antibody and FITC goat anti-rabbit IgG as secondary antibodies to distinguish them from non-myocytes. The coverslips were washed with PBS, fixed with 4% paraformaldehyde for 5 min, and infiltrated with 0.5% Triton X-100-PBS for 5 min at room temperature. Next, slips were rinsed 3 times with PBS for 3 min each and blocked with 1% BSA-PBST (PBS with 1‰ Tween-20) for 30 min at room temperature. Then NRCMs were incubated with rabbit polyclonal antibody of TNNI3 (1:500; Abclonal, A6995) at 4°C overnight. On the second day, coverslips were rinsed 3 times with PBS again and incubated with FITC goat anti-rabbit IgG (1:500, Abclonal, AS011) for 1 h. The cell nuclei were stained with DAPI for 5 min after the third time of washing with PBST. And the number of DAPI stained nuclei represented the total number of cells in the same field of microscope. Begin with the use of secondary antibodies, all subsequent operations were performed in the dark. Finally, three images were randomly captured by laser confocal microscopy for counting. And Image J was performed to count the number of myocardial cells and nuclei. The purification rate of cardiomyocytes (%) = the sum of cells labeled by fluorescence in the three visual fields ÷ the total number of cell nuclei ×100%.

### Autophagic Flux Measurement

The day before OGDR, NRCMs were transfected with Ad-mCherry-GFP-LC3 (MOI 1) for 24 h. At the end of reoxygenation, the cells were fixed with 4% paraformaldehyde, and nuclei were stained with DAPI. The fluorescence signal was observed by a confocal microscope (LEICA TCS SP8, Germany). Yellow spots (autophagosomes) and red spots (autolysosomes) under multiple visions were analyzed and counted by image J.

### siRNA-Mediated Knockdown of A2aR

To verify the role of A2aR, we used siRNA-A2aR adenovirus. siRNA sequences synthesized at Hanbio Co., LTD (Shanghai, China) were cited from the reference (31) (5′-GCUACAUCGCCAUCCGAAU-3′). Adenovirus (MOI 15) and its control vehicle were severally co-incubated with cardiomyocytes in a serum-free medium for 8 h of transfection. Next, sucking and discarding the serum-free medium, and the cardiomyocytes were continually incubated with fresh DMEM/F12 supplemented with 10% FBS, 1% penicillin-streptomycin, and 1‰ BrdU for 48 h (32). Then, immunoblot was performed to confirmed the final effect of A2aR knockdown.

### The Myocardial Ischemia-Reperfusion Injury Model

Each rat has received the anesthesia of 2% sodium pentobarbital 35 mg/kg by intraperitoneal injection (33, 34). Mechanical ventilation (Rodent Ventilator, Beijing ZSDichuang Technology Co., Ltd., China) was performed after tracheotomy. The parameters of the ventilator were adjusted as RR 60-80 beats per min, tidal volume 2 ml/100 g, the ratio of inspiration to expiration was 1:1. Open the left thorax to expose the heart. The left anterior descending coronary artery (LAD) was ligated with a 5-0 suture approximately 2 mm below the junction of the left atrial appendage and the pulmonary artery cone. Before tightening the knot, a polyethylene tube was passed through it to form an openable knot for reversible LAD occlusion. In the sham operation group, sutures only passed through the corresponding positions, and no ligation was performed. Ischemia can be affirmed by a transient drop in blood pressure and the appearance of cyanosis on the surface of myocardium. An epicardial hyperemic response and the speedy extinction of cyanosis demonstrated the recovery of reperfusion (35). After 30 min of coronary occlusion and 120 min of reperfusion (36), the rats were euthanized by injection of an overdose of anesthetic.

### Hemodynamic Measurements

After anesthesia, an electrocardiogram was connected and the right internal carotid artery (RICA) was punctured in the supine position of rats. A catheter was placed into RICA to monitor the arterial blood pressure after the anticoagulation of 100 U/kg heparin. Changes in the electrocardiogram and arterial blood pressure of rats were monitored by the BL-420 system (TaiMeng Informatization Biological Signal Acquisition and Analysis System, China). The QT interval is corrected (QTc) using Bazzet's formula to exclude the influence of heart rate (37).

### Echocardiography

Cardiac function was assessed by transthoracic echocardiography using an 11 MHz imaging transducer from GE Vivid 7 (GE Health Medical, USA). All procedures were implemented by the same researcher unknown to the experimental scheme. Parameters related to left ventricular structure: diastolic ventricular septal thickness (IVSd), systolic ventricular septal thickness (IVSs), left ventricular end-diastolic diameter (LVIDd), left ventricular end-systolic diameter (LVIDs), left ventricular diastolic posterior wall thickness (LVPWd), left ventricular systolic posterior wall thickness (LVPWs) were all obtained by M-mode ultrasound. Left ventricular end-diastolic volume (LVEDV), left ventricular end-systolic volume (LVESV), left ventricular ejection fraction (LVEF), stroke volume (SV), and left ventricular fractional shortening (FS) were automatically generated by computer algorithms.

### Measurement of Myocardial Infarction Area

After the reperfusion, the reversible LAD occlusion was completely ligated again, and 1% Evans blue dye was injected into the femoral vein. And the isolated hearts were washed three times with physiological saline, then rapidly frozen in a refrigerator at−80°C for 10 min. These isolated hearts were sliced into coronal 1-mm-thick sections by the rat heart slicer matrix (JNT-XZM, Beijing). Heart sections were soaked in 1% 2,3,5-triphenyl tetrazolium chloride (TTC) at 37°C for 15 min, and the tissues were gradually stained. The area of left ventricular area (LV) and proportions of myocardial infarction area (pale), ischemia risk area (red), and non-infarct area (blue) were measured by Image-Pro Plus 6.0 software. Ischemic area (%) = ischemia risk area (red) ÷ left ventricular size (LV) ×100%. Infarction area (%) = infarction area (pale) ÷ ischemia risk area (red) ×100%.

### Experimental Protocols

*In vitro*, each group repeated independently for 3 times (*n* = 3). And all experimental groups received 6 h of oxygen-glucose deprivation and 18 h of reoxygenation (29). CGS-21680 (A2aR specific agonist, 30μM, Tocris Bioscience, 1036) and dbcAMP (selective PKA activator, 5μM, MedChemExpress, HY-B0764) were added 1 h before reoxygenation. H89 (the PKA selective inhibitor, 10 μM, MedChemExpress, HY-15979) was used 5 min before CGS21680. To verify the effect of autophagy on cell survival, autophagy agonist Rapamycin (100 nM, MedChemExpress, HY-10219) and antagonist 3-Methyladenine (3-MA, 10 mM, MedChemExpress, HY-19312) were used 1 h before reoxygenation (38, 39).

*In vivo* study, 36 adult male rats were randomized into six groups (*n* = 6): Sham group, IR group (30 min LAD occlusion and 120 min reperfusion, 1% DMSO in 1 ml saline, iv), IR+CGS21680 group (30 μg/kg 5min before reperfusion and 30 μg/(kg·min) for 1 h, i.v.), and IR+ZM241385 group (A2aR antagonist, 0.2 mg/kg 5 min before reperfusion, i.v.), IR+dbcAMP group (5 mg/kg, 5 min before reperfusion, i.v.) (40), and IR+CGS21680+H89 group (20 mg/kg, 5 min before CGS21680, i.v.) (41).

### Protein Extraction and Western Blot

Cell and tissue samples were lysed with a RIPA lysis buffer containing protease and phosphatase inhibitory ingredients. The final protein sample was obtained after quantification by bicinchoninic acid (BCA) protein assay. Then 10% and 12% SDS PAGE gels were prepared for electrophoresis, and proteins were transferred to PVDF membranes. Finally, visualization of the target's bands was achieved using ECL-Plus detection reagents (Beyotime Biotechnology, China) and an imaging system (model 5200, Tianneng, China). The antibodies used were listed in Supplementary Table 2.

### Cell Survival Rate Detected by CCK-8 and LDH Assays

Cells were seeded in 48-well plates at a density of 9.5×10∧5/cm^2^. After 16 h of reoxygenation, 10% CCK-8 solution was added into the medium and allowed to act for 2 h to detect the absorbance at 450 nm (PerkinElmer EnSpire Microplate Reader, USA). In the blank control group, CCK-8 solution was dissolved in the cell-free medium. Given the interference of drugs and adenovirus, their control group was set as follows: adding agonist, antagonist, or adenovirus to cell-free medium respectively, and then adding CCK-8 solution. According to the instruction, the OD value was calculated as the cellular viability. The level of LDH (lactate dehydrogenase), an indicator of cell necrosis, was measured using the kit following its operating instructions (Elabscience, E-EL-R0338c). The final absorbance was scaled at 450 nm.

### The Concentration Measurement of CK-MB and cTnI

When ending reperfusion, the medium samples of each group were collected, centrifuged at 12,000 rpm for 1 min, and the supernatant was sucked out to detect the concentration of CK-MB and cTnI. Indicators of cardiomyocyte injury, CK-MB (creatine kinase-MB, Elabscience, E-EL-R1327c) and cTnI (cardiac troponin I, Elabscience, E-EL-R1253c), were detected by the corresponding kits followed its instruction. The final absorbance was scaled at 450 nm.

### Hematoxylin-Eosin Staining

The hearts were harvested rapidly after reperfusion and washed by sterile normal saline 3 times. Next the hearts were fixed by paraformaldehyde for 24 h. And the fixed heart was successively treated with ethanol dehydration (the concentration gradient of 70%, 80%, 90%, 95%, and 100%), xylene transparency and paraffin embedding. Then, the paraffin tissue blocks were prepared as 5 μm sections. Tissue sections were progressively subjected to xylene dewaxing, ethanol hydration (the concentration gradient of 100%, 95%, 80%, and 75%), and hematoxylin-eosin staining. Last, all tissue slices were dehydrated, transparentized, and sealed with neutral resins. The myocardial pathological alterations of the slices were assessed by the optical microscope.

### Transmission Electron Microscopy

After reoxygenation, myocardial cells were collected and fixed with 2.5% glutaraldehyde at 4°C for 4 h. Next, cells were rinsed 3 times with 0.1M phosphoric acid buffer, and fixed with 1% osmic acid for 2 h. The samples were dehydrated in gradient ethanol (50%, 70%, 90%, 100%), permeated, and embedded in epoxy resin at 60°C for 48 h. Then the embedded samples were sliced into 80 nm by ultrathin slicer (Leica, EM UC7, German) and stained with 2% uranyl acetate and lead citrate. The morphological structure and autophagy flux of cells was observed by the transmission electron microscope (Hitachi TEM system, HT7800, Japan). *In vitro* experiment, random observation was performed on three cardiomyocytes in each group and the images were taken in a clockwise direction around the nucleus. The number of autophagosomes and autolysosomes was counted at 10000 magnification times. In animal experiments, a field of view was stochastically chosen from each rat's sample to count the quantity of autophagosomes and autolysosomes.

### Statistical Analysis

All data were expressed as mean ± standard error of the mean unless otherwise specified, and statistical differences between groups were analyzed by GraphPad Prism 8.0 (GraphPad Software, Inc., La Jolla, CA). And the differences between multiple groups were verified through the one-way ANOVA with Bonferroni or Dunnett *post-hoc* test unless otherwise stated. Data, passed the normality and equal variance tests, were normalized to the control or sham groups. A *p*-value of <0.05 was considered statistically significant.

## Results

### A2aR's Activation Reduced QTc Prolongation and Facilitated the Recovery of Ventricular Systolic Function After Ischemia

**Figure 1:**
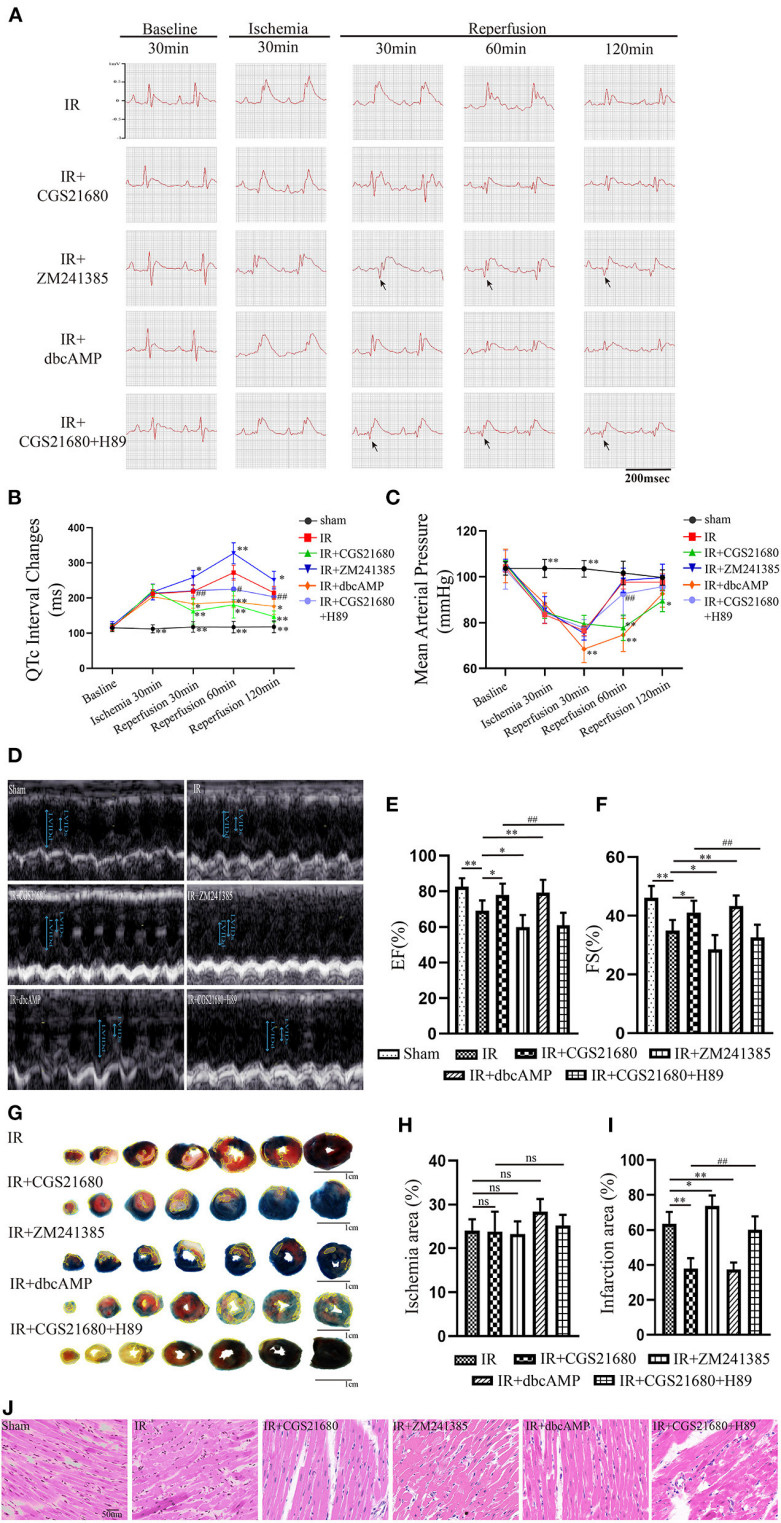
A2aR's activation facilitated the recovery of ventricular systolic function after ischemia. **(A,B)** Real-time ECG and the QTc interval change in different time points. Arrows showed the pathological Q waves. **(C)** Hemodynamic parameters during the experiment. **(D)** Ultrasound M-mode images of the parasternal long axis showed left ventricular motions. The examination was performed on animals with the left lateral decubitus position. **(E,F)** LVEF, and LVFS were calculated by echocardiography. Data presented as mean ± SD and analyzed by one-way ANOVA with uncorrected Fisher's LSD *post hoc* test. **(G)** Evans blue-TTC staining of cardiac tissue. The infarct area was marked with yellow lines. **(H,I)** Analysis of the ischemic and infarct area of the ventricle. **(J)** HE staining of cardiac tissue. **P < 0.01, *P < 0.05, and ns means *P* > 0.05 vs. IR group. ^*##*^*P*<0.01, ^#^*P*<0.05 vs. IR+CGS21680 group. Unless otherwise noted, all data presented as mean ± SD and analyzed by one-way ANOVA with Bonferroni *post hoc* test (*n* = 6).

To reassess the effect of A2aR on cardiac function in MIRI, we established the model of rats i*n vivo*. The ST-segment elevation of ECG indicated a successful establishment of the ischemic model. MIRI caused the QTc prolongation of the ECG. From the point of 30 min' reperfusion to the end, the ECG of the A2aR antagonist group appeared inverted Q waves with larger amplitude (as showed by arrow) and had a longer QTc interval. On the contrary, the prolongation of QTc interval was significantly improved after A2aR activation (Figures 1A,B). Moreover, we monitored the carotid artery pressure of each rat in real time after anesthesia. In order to explore the impact of drug treatments on the hemodynamics of rats in each group, we selected 5 time points (Figure 1C). The mean arterial pressure (MAP) of the Sham group always remained stable at about 100 mmHg. The ischemic treatment of the three experimental groups significantly decreased the MAP, and after 30 min of reperfusion, the decline became more visible. But the MAP among the three experimental groups were not statistically significant at the point of reperfusion 30 min. At 60 min of reperfusion, rats in the IR group and IR+ZM241385 group had adapted to reperfusion. And their MAP returned to a level of about 100 mmHg back. Oppositely, the MAP of the A2aR agonist group decreased further than before at 60 min of reperfusion, and it kept the low level until the end of reperfusion.

Five min before the end of reperfusion, we performed a cardiac ultrasound on each rat to investigate if its cardiac function had changed (Figure 1D). And echocardiography showed that ischemia-reperfusion injury impaired left ventricular ejection function (LVEF) and fractional shortening (LVFS) (Figures 1E,F). LVEF and LVFS in the IR+CGS21680 group improved evidently, while those in the antagonist ZM241385 group decreased further. The traditional Evans Blue-TTC staining method was performed to calculate the size of myocardial infarction. Based on the IR group, the infarct area of the IR+CGS21680 group decreased by 41%, while that of the ZM241385 group was conversely increased (Figures 1G,I). The ischemic areas among the groups were not statistically significant, indicating that the LAD ligated sites were consistent in the model (Figure 1H). These demonstrated that A2aR activation before reperfusion contributed to ameliorate ventricular systolic function and lessen the area of myocardial infarction.

### A2aR's Activation Inhibited Autophagy and Apoptosis Produced by MIRI and Diminished Myocardial Cell Death

**Figure 2:**
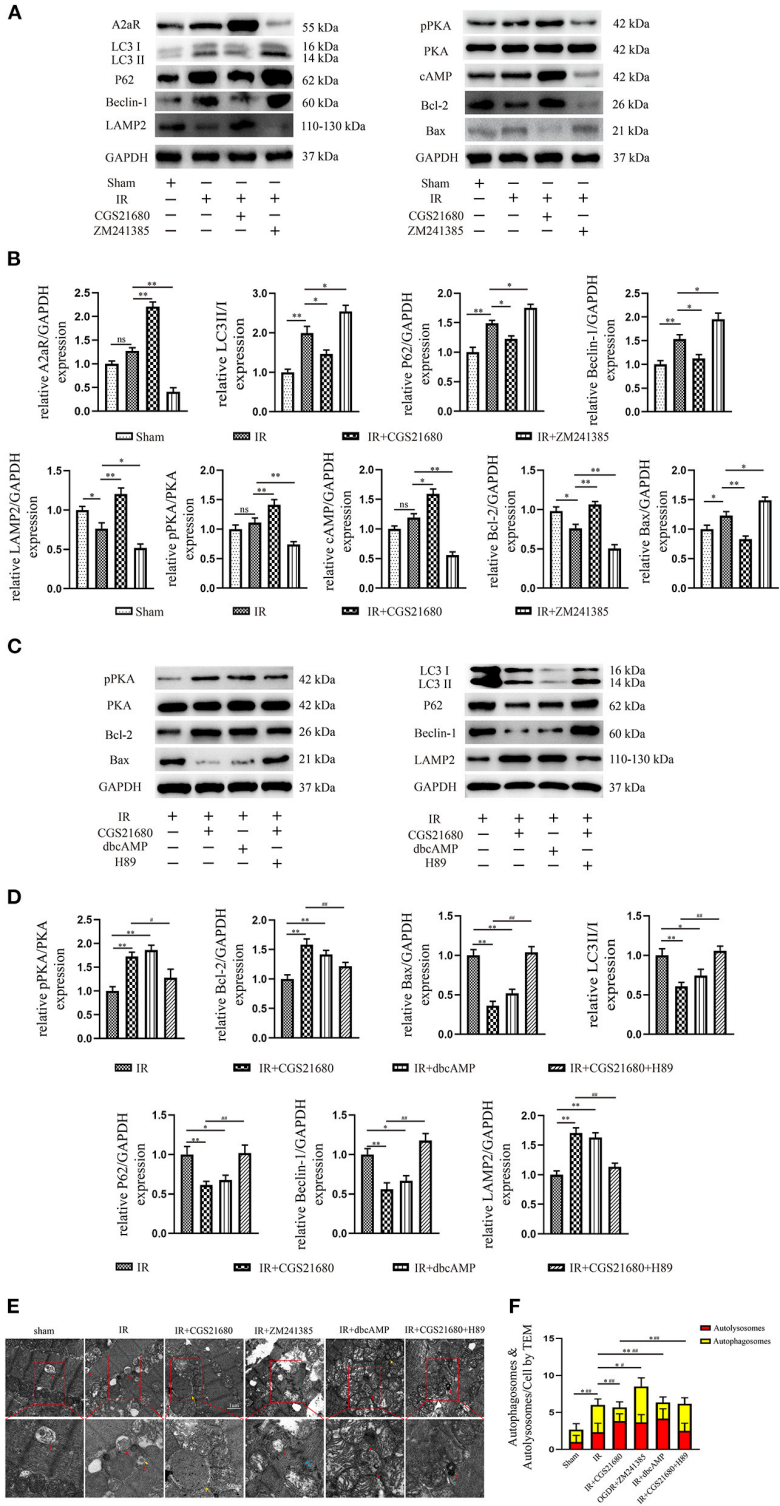
A2aR's activation inhibited autophagy and apoptosis caused by MIRI. **(A,C)** Representative immunoblotting results of autophagy and apoptosis-related protein. **(B,D)** All data of western blot were presented as mean ± SEM and analyzed by one-way ANOVA with Bonferroni *post hoc* test. *P < 0.05, **P < 0.01, and ns means *P* > 0.05 vs. IR group and ^#^*P* < 0.05, ^*##*^*P* < 0.01 vs. IR+CGS21680 group. **(E,F)** The morphological changes of rat hearts were observed under the transmission electron microscope. ^#^*P* < 0.05, ^*##*^*P* < 0.01 represented the statistical significance between two groups in autophagosomes' number. *P < 0.05, **P < 0.01 represented the difference between two groups in autolysosomes. All data presented as mean ± SD and analyzed by one-way ANOVA with Bonferroni *post hoc* test; *n* = 6.

To explore the change brought by ischemia-reperfusion injury (IR), we performed immunoblotting. The results manifested that IR could induce the high level of autophagy and apoptosis as the increased LC3II/I, P62, Beclin-1, and Bax, while the antiapoptotic protein Bcl-2 and lysosome membrane protein LAMP2 oppositely decreased (Figures 2A,B). Although A2aR showed an increasing trend in the IR group, that was not statistically significant compared with the Sham group. When A2aR was significantly enhanced with CGS21680, the expression of LC3II/I, P62, Beclin-1, and Bax was decreased, whereas Bcl-2 and LAMP2 increased. On the contrary, autophagy and apoptotic production in the antagonist ZM241385 group were significantly increased. Furthermore, consistent with the change level of A2aR, the expression of cAMP and p-PKA increased significantly after CGS21680 stimulation but decreased remarkably after ZM241385 stimulation.

Changes in myocardial tissue structure were investigated by HE staining. The myocardial fibers in Sham group were neatly arranged and compact (Figure 1J). In the IR group, however, the fibers were broken and wavy, accompanied by inflammatory cell infiltration and erythrocyte exudation. Cardiomyocytes swelling, myofibrillar fracture, and cell nuclei blur were exacerbated in the antagonist ZM241385 group. Inversely, the muscle fiber breakage was significantly decreased and the fiber texture was visible in the A2aR agonist group.

Transmission electron microscopy (TEM) was one of the most convincing methods to observe and evaluate autophagy flow. Myocardial fiber texture and Z-line were clearly visible in the Sham group (Figure 2E). Mitochondria with normal morphology and cristae structure were arranged between myocardial fibers. And a small amount of basic autophagy flow was observed under normal physiological conditions (red arrow indicated the autolysosome). In the IR group, mitochondria were swollen, vacuolar degeneration and the crest structure were damaged and blurred. The emergence of autophagosomes and autolysosomes increased, but the amount of autophagosomes was dominant (Figure 2F). There are more megamitochondria and vacuoles in the IR+ZM241385 group, accompanied by the rupture and dissolution of muscle fibers and intercalated disk (indicated by blue arrow). Consistent with the severity of tissue injury, more autophagosomes were produced and the autophagy flux was blocked in this group. In contrast, the autophagy flux was apparently restored with increased autophagy-lysosome fusion when A2aR was activated. Meanwhile, the myocardial damage was significantly improved, manifested by reduced vacuolar degeneration and disruption of mitochondrial cristae. These results suggested that A2aR plays a protective part in the myocardium by inhibiting autophagy and apoptosis.

### The cAMP-PKA Signaling Pathway Was Participated in the Cardioprotection of A2aR

Western blot revealed the A2aR's downstream signal cAMP and PKA was changed under IR. Hence, the special agonist and antagonist of PKA were chosen to test the function of cAMP-PKA signal pathway in MIRI. The agonist IR+dbcAMP group effectively ameliorated IR-induced QTc prolongation at 30, 60 and 120 min of reperfusion. After using dbcAMP, the MAP in this group showed a significant downward trend. But it began to rise gradually after 30 min of reperfusion, and till the finish of reperfusion, there was no significant difference with the IR group (Figures 1B,C). Consistent with the ECG performance of the IR+ZM241385 group, the antagonist IR+CGS21680+H89 group also showed inverted Q waves, and the QTc interval was prolonged at 30 min of reperfusion. At 60 min of reperfusion, MAP in the antagonist IR+dbcAMP group was statistically higher than in its control IR+CGS21680 group, but not significantly different at other time points (Figures 1A–C). Echocardiography and tissue staining showed that downstream PKA activation with dbcAMP was equally effective in improving IR-induced impairment of EF and FS and reducing myocardial infarct size (Figures 1D–I). And HE staining showed a neat arrangement of myocardial fibers with clear nuclei and fewer breakage in this group (Figure 1J). However, the antagonist H89 counteracted the cardioprotective effects of CGS21680 during the reperfusion phase, manifested by decreased EF and FS, markedly swollen and ruptured myocardial fibers, and increased myocardial infarct size (Figures 1D–J).

Western blot analysis displayed that the addition of cAMP analog, dbcAMP, promoted the phosphorylation of PKA, the expression of LAMP2, and the level of anti-apoptotic protein Bcl-2. In contrast, the production of autophagy and apoptosis-related proteins, Beclin-1, P62, LC3II, and Bax, were significantly inhibited (Figures 2C,D). Moreover, using transmission electron microscopy (TEM) to observe autophagic flow found that, the use of dbcAMP promoted autophagosome-lysosome fusion, alleviated mitochondrial edema and cristae damage, and significantly reduced vacuolar degeneration (Figures 2E,F). These results suggested that the activation of A2aR's downstream cAMP-PKA had the same inhibitory effect on autophagy and apoptosis as activation of A2aR. When the downstream cAMP-PKA signaling pathway of A2aR was blocked by H89, however, the myocardial protective effect of A2aR was eliminated. Protein synthesis associated with autophagy initiation in the IR+CGS21680+H89 group, such as Beclin-1, P62, LC3II, was significantly increased, while lysosomes process autophagosomes-related LAMP2 was inhibited. The level of pro-apoptotic Bax was also enhanced, but the anti-apoptosis Bcl-2 was conversely decreased (Figure 2E). Furthermore, the blockage of autophagic flux caused the accumulation of autophagosomes under TEM (Figure 2F). And myocardial muscle fibers showed dissolution and vacuolar degeneration.

Overall, the animal experiment with MIRI manifested that A2aR activation can reduce cell death caused by autophagy and apoptosis, improve left ventricular systolic dysfunction, and diminish myocardial infarction. Moreover, this protective effect of A2aR was related to the cAMP-PKA signaling pathway.

### Purification of Cardiomyocytes *in vitro*

**Figure 3:**
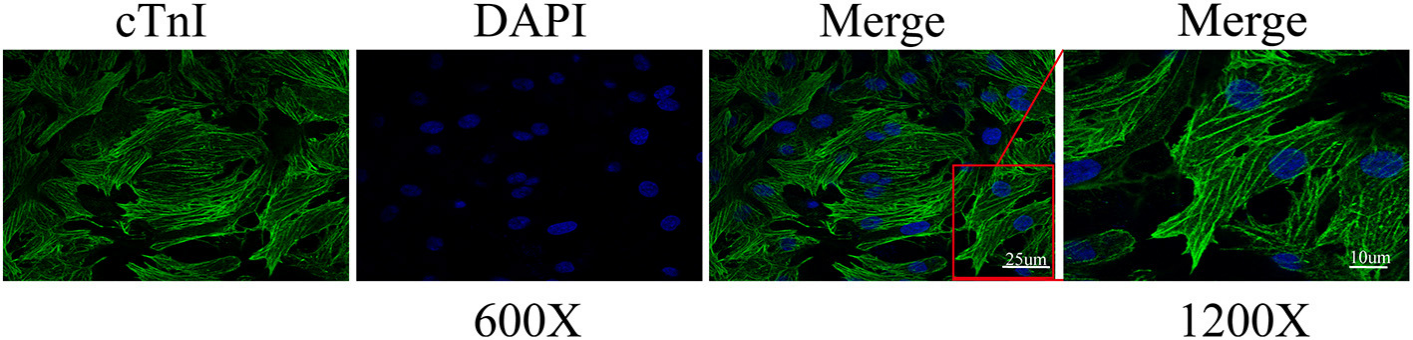
Cardiomyocyte identification by cardiac troponin I (cTnI). The representative immunofluorescence images were captured by confocal microscopy at 600 and 1200× magnification. Calculation by image J, the ratio of the number of cardiomyocytes to total cells of three different sights was 97.8%.

To verify whether A2aR had the same effect *in vitro* models, we extracted primary myocardial cells. In cell extraction, the final cell suspension contained vascular endothelial cells, smooth muscle cells, and ventricular muscle cells. Given the non-dividing and proliferative characteristics of cardiomyocytes, we improved their purification rate by the centrifugal selection, differential paces of sticking to the wall, and BrdU drug inhibition. After 24 h of incubation, we observed the rhythmic beating of cardiomyocytes under a microscope. And cardiomyocytes were labeled with a specific antibody of cardiac troponin I (cTnI). Under confocal microscopy (600× and 1200×), the sarcomere of cells appeared as the striations of light and dark (Figure 3). Eventually, the purification rate of cardiomyocytes reached 97.8%. Therefore, in the following experiments, the interference of non-muscular cells was no longer considered.

### Increased Autophagy Caused Cardiomyocyte Death Under OGDR

**Figure 4:**
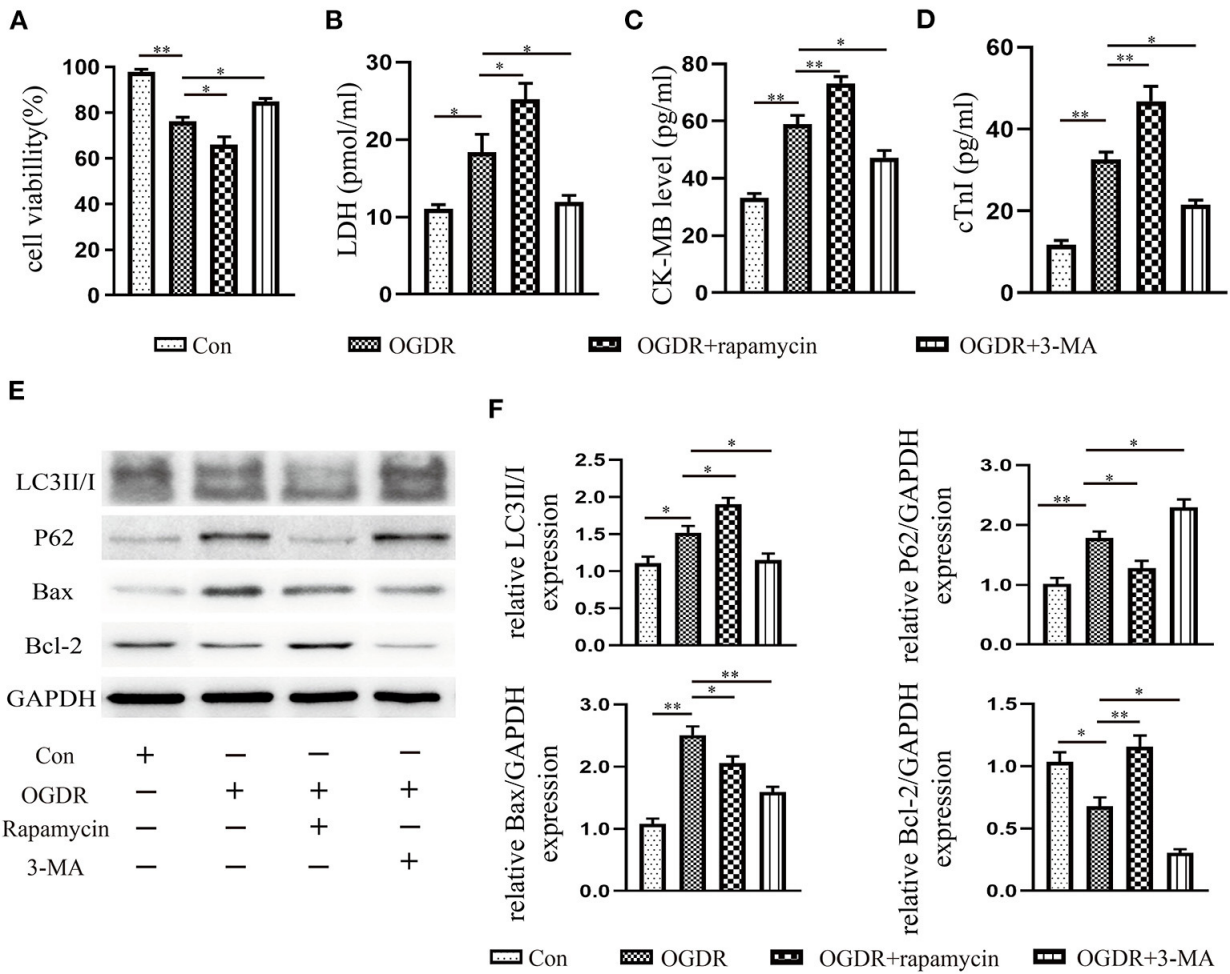
Increased autophagy caused cardiomyocyte death under OGDR. **(A)** Cell viability was assessed by the CCK-8 reagent. **(B–D)** LDH, CK-MB, and cTnI released from cardiomyocytes were detected by the Elisa kit. **(E,F)** Representative western blot results, and image J analysis results of LC3II/I, P62, Bax, and Bcl-2. All data presented as mean ± SEM and analyzed by one-way ANOVA with Dunnett's *post hoc* test; *n* = 3. **P < 0.01, *P < 0.05 vs. OGDR group.

An OGDR model *in vitro* was used to simulate ischemia-reperfusion injury *in vivo*. Compared with the control, the cell survival rate was deteriorating rather noticeably in the OGDR group (Figure 4A). The level of LDH, CK-MB, and cTnI releasing from dead and damaged cells prominently increased (Figures 4B–D). Then western blot showed that OGDR triggered autophagy and apoptosis in cardiomyocytes, as evidenced by a remarkable increase in the level of LC3II/I, P62, and Bax (Figures 4E,F). To clarify the relationship between autophagy and apoptosis, we used an autophagy antagonist and agonist before reoxygenation. Rapamycin (100 nM) stimulated the increase of LC3II and the depletion of its substrate P62. Although the anti-apoptotic protein Bcl-2 increased in response to rapamycin, the cell survival rate still declined. And the release of CK-MB, cTnI, and LDH was further increase. In contrast, cell death was improved until autophagy was suppressed by 3-MA (10 mM). And the apoptosis also recovered with the reduction of autophagy. These suggested that over-activated autophagy during reperfusion had an anti-apoptotic effect and was harmful to cell survival.

### The Protection of A2aR in Attenuating OGDR-Induced Cell Death Depended on Restraint to Apoptosis and Autophagy

**Figure 5:**
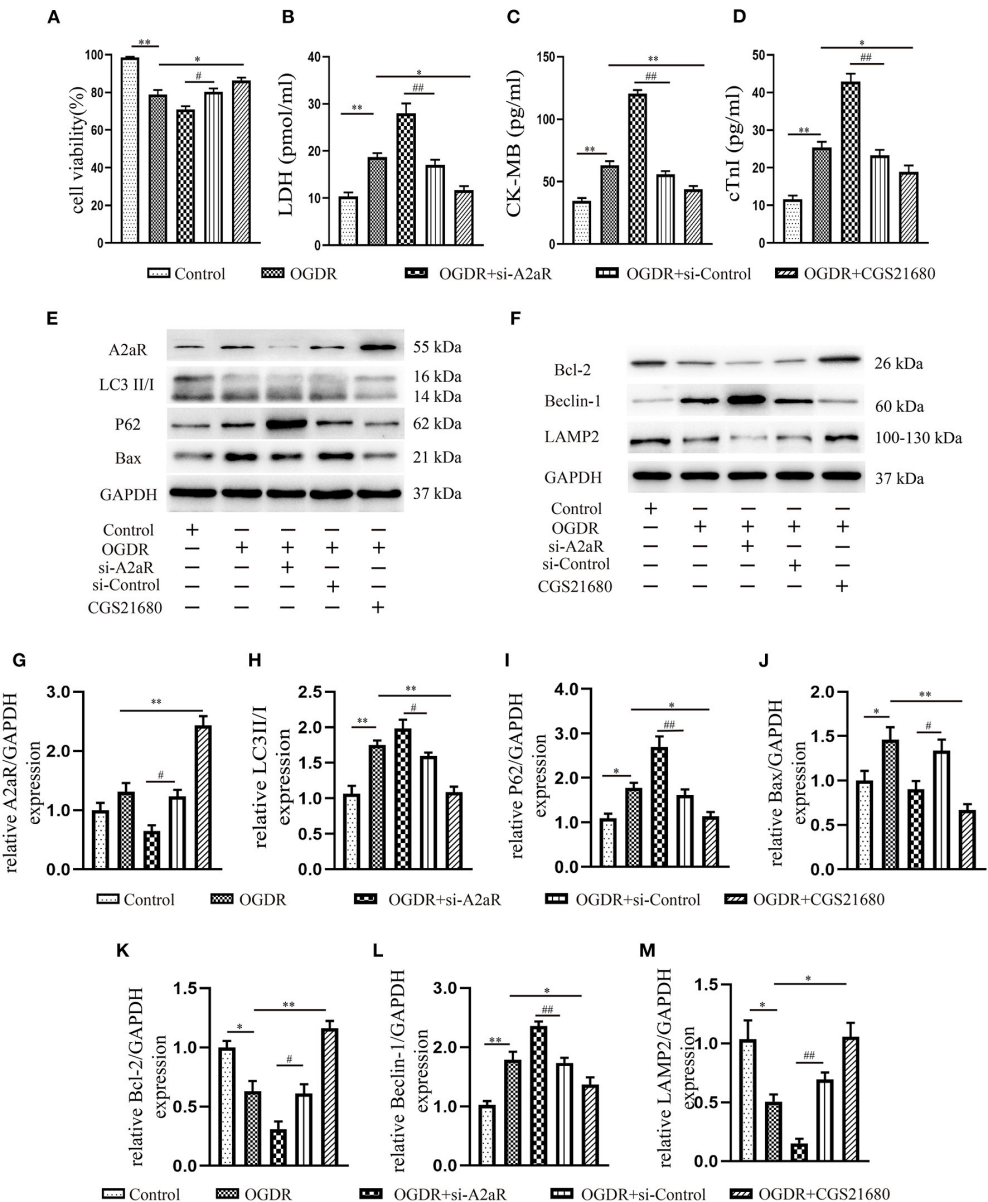
The protection of A2aR in attenuating OGDR-induced cell death depended on inhibition of apoptosis and autophagy. **(A)** Cell viability was detected by CCK-8. **(B–D)** LDH, CK-MB and cTnI in the medium were measured by Elisa kit. **(E–M)** The content of related proteins was evaluated by western blot and then analyzed by image J. *P<0.05, **P < 0.01 vs. OGDR group and ^#^*P* < 0.05, ^*##*^*P* < 0.01 vs. OGDR+si-Control group. All data presented as mean ± SEM and analyzed by one-way ANOVA with Dunnett's *post hoc* test; *n* = 3.

To investigate the role of A2aR in OGDR damage, CGS21680 was used to activate A2aR 1 h before reoxygenation. In the agonist group, cell survival visibly improved (Figure 5A), and fewer cytoplasmic components, such as LDH, CK-MB, and cTnI released into the culture medium (Figures 5B–D). And CGS21680 inhibited the level of Bax and the conversion of LC3I to LC3II (Figures 5E,H,J). The reduced substrate protein P62 of the autophagosome also confirmed the reduction of autophagy production (Figures 5E,I). Besides, A2aR activation increased Bcl-2 expression, decreased the level of Beclin-1, and further promoted the expression of LAMP2 (Figures 5F,K–M). To verify that, we knocked down A2aR expression with adenovirus of siRNA-A2aR (Figures 5E,G). In the OGDR+si-A2aR group, however, the level of LDH, CK-MB, and cTnI was respectively increased 1.65 times, 2.16 times, and 1.84 times compared with the si-Control group. And accompanied by those increased indexes, the cell viability decreased significantly (Figures 5A–D). Gene knockdown of A2aR can increase the expression of Bax, LC3II and Beclin-1 while inhibiting the production of LAMP2. A decline in the expression level of the lysosome membrane protein, LAMP2, represented impaired autophagosome processing. These proved that activation of A2aR can inhibit apoptosis and autophagy, thus saving cardiomyocytes for survival.

### A2aR′s Cardioprotective Effect Was Modulated via the CAMP-PKA Signaling Pathway

**Figure 6:**
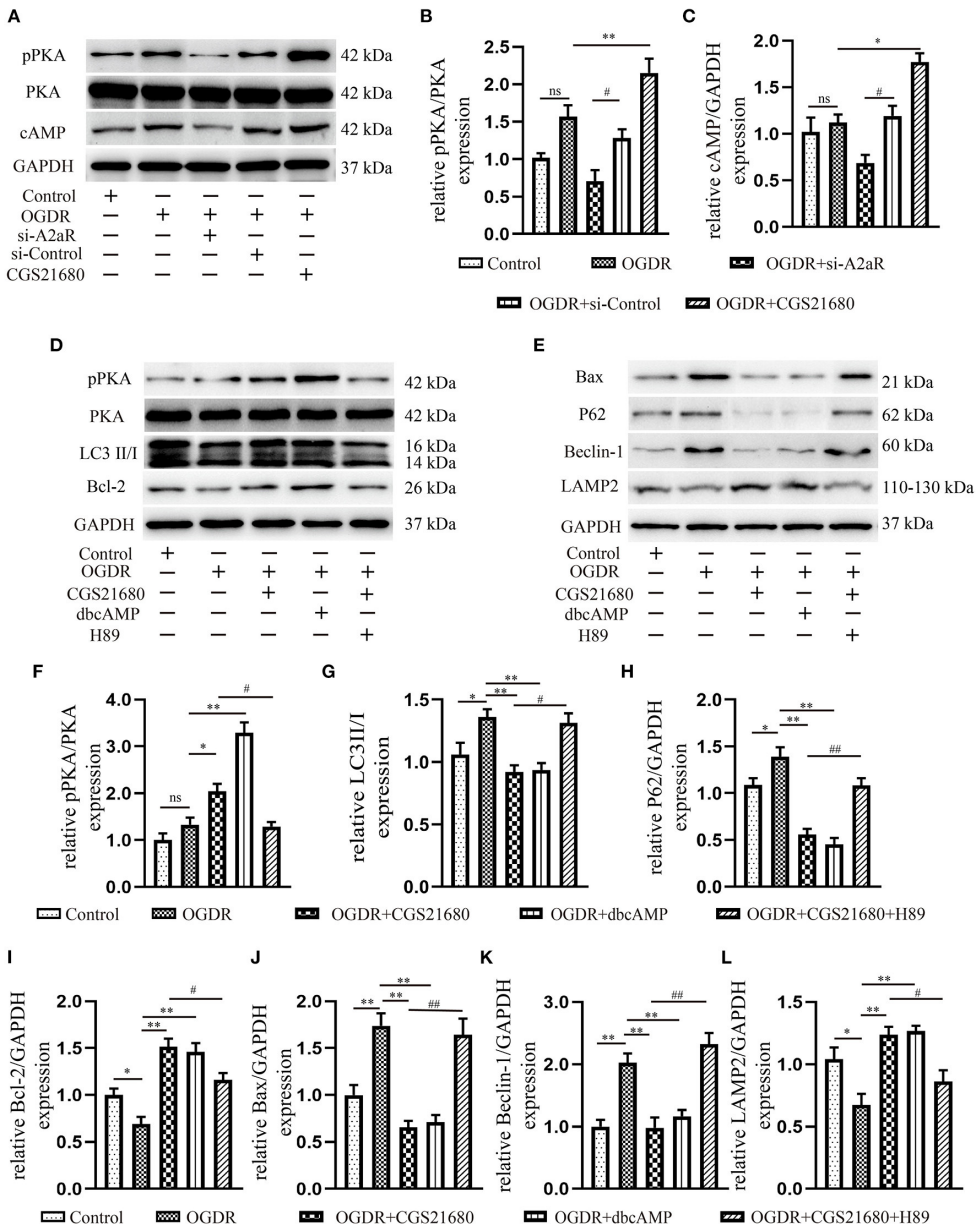
A2aR′s cardioprotection was mediated via the cAMP-PKA signaling pathway. **(A–C)** Quantitative analysis of the corresponding protein. *P < 0.05, **P<0.01 vs. OGDR group and ^#^*P* < 0.05, ^*##*^*P* < 0.01 vs. OGDR+si-Control group. **(D–L)** Expression of respective target protein and the quantitative analysis by image J. *P < 0.05, **P < 0.01 vs. OGDR group and ^#^*P* < 0.05, ^*##*^*P* < 0.01 vs. OGDR+CGS21680 group. All data presented as mean ± SEM and analyzed by one-way ANOVA with Bonferroni *post hoc* test; *n* = 3.

Consistent with the results of animal experiment, A2aR and its downstream cAMP and p-PKA showed an increasing trend under OGDR, but there was no statistical significance. When given an additional agonist, the increase of the A2aR level could raise the level of intracellular cAMP and promote the phosphorylation of PKA. si-A2aR, however, diminished intracellular cAMP and p-PKA expression (Figures 6A–C). A selective PKA activator, dbcAMP, was used to test the effect of cAMP-PKA in A2aR-induced protection. It showed dbcAMP did the same as A2aR in the restraint of autophagy generation and anti-apoptosis effect (Figures 6D–L). Reversely, the PKA selective inhibitor, H89, can significantly eliminate the myocardial protection of A2aR. Thus, these results concluded that the cAMP-PKA signaling pathway participated in the cytoprotective action of A2aR.

### The Protective Effect of A2aR Relied on the Inhibition of Autophagosome Generation

**Figure 7:**
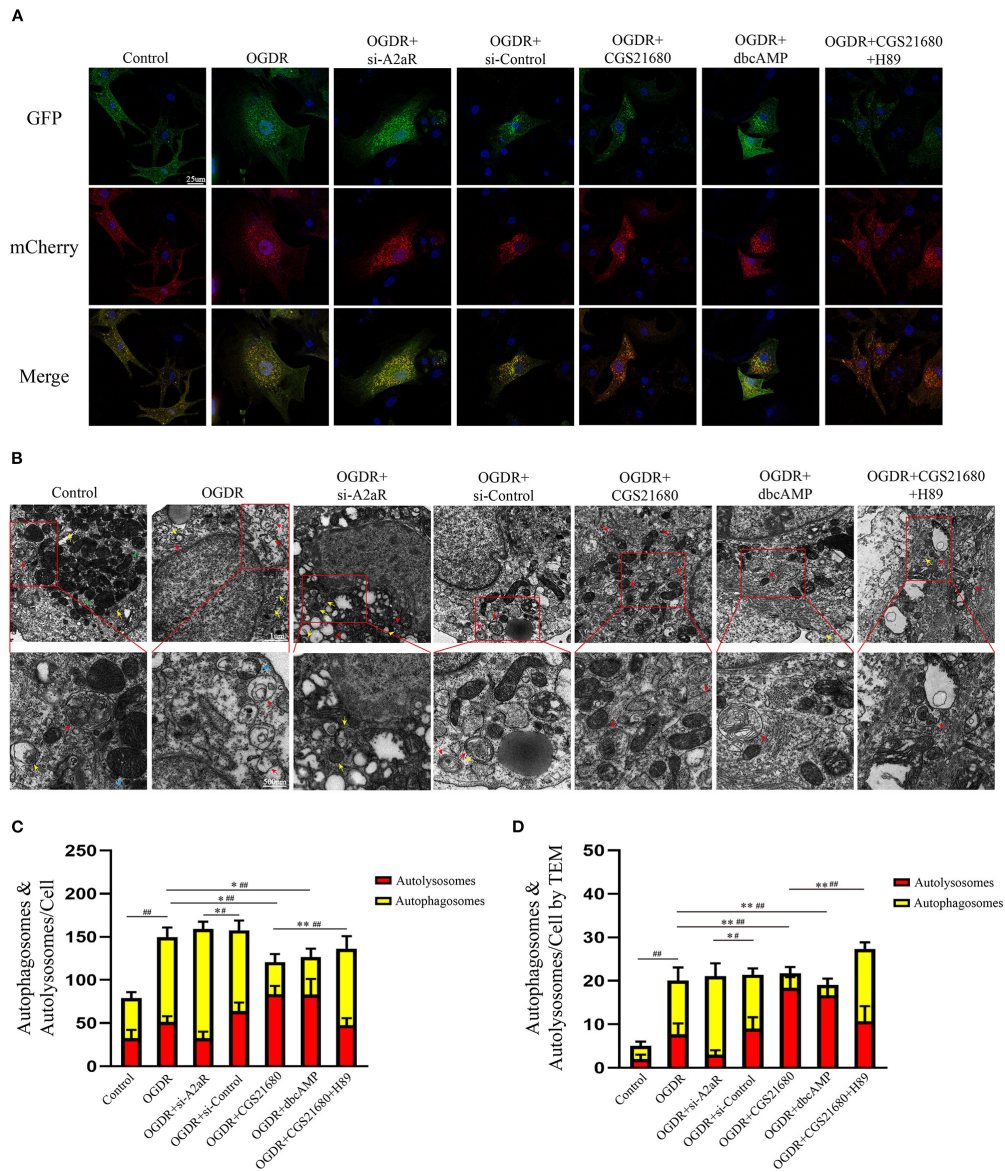
The protective effect of A2aR relied on the inhibition of autophagosome generation. **(A,C)** Autophagy flux detected by Ad-mCherry-GFP-LC3B. Representative graphs (630×) of each group under a confocal microscope and quantitative analysis of red and yellow spots in multiple fields. **(B,D)** Transmission electron micrographs of autophagosomes (indicated by yellow arrows and triangles) and autolysosomes (indicated by red arrows). Blue arrows indicate myocardial fibers, and green arrows indicate mitochondria. ^#^*P* < 0.05, ^*##*^*P* < 0.01 represented the statistical significance between two groups in autophagosomes' number. *P < 0.05, **P < 0.01 represented the difference between two groups in autolysosomes. All data presented as mean ± SD and analyzed by one-way ANOVA with Bonferroni *post hoc* test; *n* = 3.

The mCherry-GFP-LC3II adenovirus was transfected NRCMs to study the changes of autophagy flux. Compared with the control group, yellow autophagosomes in the OGDR group were significantly increased and were dominant in terms of number. Red autolysosomes in the OGDR group had an increasing trend, but there was no statistically significant compared with the control group (Figures 7A,C). After A2aR or its downstream PKA activation, the formation of autophagosomes was inhibited, and autophagy flux recovered. Thus, autolysosomes were significantly dominant in counting. In contrast, when siA2aR or PKA antagonist H89 was used, the autophagic flow was further impaired, and the autophagosomes accumulated in cells and were difficult to degrade.

The autophagy flux of NRCMs was also observed by TEM (Figures 7B,D). In the control group, filamentous myocardial muscle fibers can be observed (as shown by green triangle, Figure 7B). There were plenty of normal mitochondria with clear crest structure around the nucleus (blue arrow). And a small amount of autophagic flux (yellow arrow represented autophagosome, red arrow represented autolysosome) was observed under normal physiological conditions. In the OGDR group, the number of autophagy and autolysosome was both increased in puff cytoplasm (42). Autophagosome was a double-membraned vesicle (yellow arrow), and autolysosome was single-membraned vesicle (red arrow) containing ruined organelles, cellular debris, and membrane-like structures (43, 44). The yellow triangle represented the autophagosome surrounded by autolysosome. In addition, OGDR damaged the mitochondrial crest structure, which was fractured and blurry (blue arrow). In si-A2aR group, cells were showing perinuclear cytoplasmic vacuolation and mitochondrial blebbing compared to its si-control group. And the number of its autophagosomes correspondingly increased. Interestingly, the activation of A2aR or its downstream PKA recovered the autophagic flux after OGDR by promoting autophagosome conversion to autolysosome (Figure 7D). Cell damage caused by OGDR in those two groups was also reduced, as evidenced by decreased mitochondrial vacuolation and clear crest structure. Notably, megamitochondria with fractured crest structure appeared in the OGDR+CGS21680+H89 group, and autophagy flow was also impaired. These results from the perspective of confocal immunofluorescence and TEM were consistent with the western blot ones.

## Discussion

CHD remains the leading mortality among diseases around the world, whose root cause is the death and loss of non-proliferative myocardial cells. And most cell death occurs during ischemia-reperfusion (45). Cell death involved in MIRI manifests in various forms, specifically apoptosis, autophagy, pyroptosis, and ferroptosis (46). Morphologically, pyroptosis has the manifestations as apoptosis (DNA fragmentation, nuclear condensation) and necrosis (cellular swelling, formation of pores in the cytomembrane, and rupture of cells). Mechanistically, pyroptosis is characterized by its dependence on inflammatory caspases (mainly caspase-1, 4, 5, and 11) and along with the release of pro-inflammatory factors (45, 47). Studies have shown that cardiomyocytes under IR showed classic morphological features of pyroptosis: cell swelling, formation of bubble-like protrusions, formation of pores in the cytoplasmic membrane by gasdermin D (GSDMD), rupture of the cell membrane, and the release of inflammatory factors IL-1β and IL-18 (48). There is a positive correlation between pyroptosis and MIRI severity by existing research. Conversely, inhibiting the production of NLRP3 inflammasome or GSDMD induced pyroptosis can reduce MIRI and myocardial infarction size (49, 50).

Ferroptosis, a new type of iron-dependent regulatory cell death, is distinguished from apoptosis, necrosis, pyroptosis, and autophagy in morphology. The distinct feature of ferroptosis is principally represented as mitochondrial variation, covering mitochondrial shrinkage, membrane densification, and cristae damage. Whereas, the morphology of cell nucleus is generally normal (51). Multiple signal pathways and metabolic reactions are involved in the occurrence of ferroptosis, which is a complex mechanism. The primary mechanism of ferroptosis is considered as the system ${\text{x}}_{\text{c}}^{-}$/GSH/GPX4 pathway of amino acid metabolism. Furthermore, iron metabolism and lipid metabolism are also the essential formation condition for ferroptosis (52). Ferroptosis has been confirmed to be involved in MIRI and has become a new therapeutic target of MIRI in recent years (53). Inhibiting transferrin function, reducing intracellular Fe^2+^ deposition, and anti-lipid peroxidation are all effective means to reduce ferroptosis and improve MIRI (54–57).

The patterns of cell death in MIRI are diverse and complicated, and each of the programmed cell death is interrelated and interactive to others. This study focused on exploring the mechanism of action of autophagy and apoptosis in MIRI.

Autophagy is an intracellular catabolism process that is a beneficial process of energy recovery and reuse. For non-dividing cells such as myocardium, autophagy activation, which can provide amino acids, fatty acids, ATP, or other energy substrates, is especially essential for cell survival and normal tissue function during ischemia or nutrient deficiency. And, autophagy is a considerable regulator to maintain the stable structure and stable functionality of the heart. Under the stimulation of stress, protein aggregates increase and accumulate in the cytoplasm, which is usually malignant for cell survival. P62 identifies those aggregates and recruits LC3II (58, 59), forming autophagosomes with the phagophore. Then, autophagosomes and lysosomes fuse to form autolysosomes with the help of a crucial factor, LAMP2 (60, 61). And autolysosomes break down damaged organelles and harmful proteins, making cells survive.

However, this stress-induced autophagy appears to be beneficial only when activated at the right time and to the appropriate level. Our study found that the OGDR-induced autophagy resulted in cardiomyocytes' damage and decreased survival rate characterized as the release of LDH, CK-MB, and cTnI. We observed that OGDR caused the cumulation of numerous autophagosomes in cells, while the increase of autolysosomes was not significant through the fluorescent tags of LC3II and TEM. These were because autophagy during reperfusion stimulated by the Beclin-1-dependent mechanism (23) and the rise of Beclin-1 in OGDR suppressed the expression of LAMP2, which caused impaired production of autolysosomes and accumulated autophagosomes. Moreover, the overactive autophagy with rapamycin aggravated cell death, and it also played an anti-apoptosis role. When autophagy was inhibited, however, the cells exhibited apoptosis of Bax depletion. It was consistent with the views (23, 62) that excessive autophagy during the reperfusion was detrimental. Also, it indicated a contradictory relationship between autophagy and apoptosis under the OGDR condition.

Autophagy and apoptosis are two different adaptive responses of cells under stress. The relationship between autophagy and apoptosis is intricate and variable, which ultimately determines cell fate. Some protein interactions bridge the link between autophagy and apoptosis. It has been confirmed that the anti-apoptotic protein Bcl-2/Bcl-X(L) can inhibit autophagy by binding to the Bcl-2 homology-3 (BH3) receptor domain of Beclin-1 (63). The Beclin-1-Bcl-2/Bcl-X(L) complex is critical to MIRI, for its vital role in transforming autophagy and apoptosis in the cell (24). Our results suggested that post-treatment with activation of A2aR may alleviate MIRI through promoting anti-apoptotic Bcl-2 production,inhibiting Beclin-1 expression, and reducing autophagosome formation. It enabled cell survival, promoted the recovery of cardiac contractility, and reduced the area of infarction. Inversely, after knocking down A2aR, cumulate autophagosomes exasperated cell death and myocardial infarction injury. The results differ from previous studies (7), possibly because the duration of the agonist's action varies. However, it is worth noting that continuous pump injection of CGS21680 and the use of dbcAMP may cause a significant drop in blood pressure levels. This hypotension side effect was associated with bradycardia and vasodilation caused by massive activation of A2aR and cAMP (4). Conversely, despite more severe myocardial damage, the compensatory effect of the fast heart rate kept blood pressure stable in the A2aR and PKA antagonist group.

In the classical signal cascade reaction, adenosine or its analogs can activate AC after activating A2aR, causing an increase of cAMP and PKA. The cAMP-PKA pathway has a protective effect on IRI in multiple organs, for instance, the brain (64), intestine (65), liver (66), kidney (67), and heart (7). For MIRI, the participation of cAMP and PKA in OGDR may explain the down-regulation of autophagy. It involves two aspects, one of which is the direct regulation of autophagy by cAMP-PKA. Increased PKA activity will restrict harmful autophagy, whereas the deactivation of PKA will induce a strong autophagy response (68), as evidenced by our use of its activators and inhibitors. The reason is that the Atg1/Atg13 complex, the key to signal integration in the autophagy pathway, is the direct substrate of PKA (69). And cAMP-dependent PKA can regulate Atg1 phosphorylation, thereby regulated the early autophagosome formation (70) and inhibiting the occurrence of autophagy (71). Also, PKA can inhibit autophagy by phosphorylating the Ser12 site of LC3 (58). And the other aspect could be the involvement of Beclin-1-Bcl-2 related mechanisms. The study of Wang et al. (72) showed that the level of Beclin-1 expression determines the detrimental or beneficial action of autophagy activity. Our results indicate that activations of both A2aR and PKA could inhibit the high levels of Beclin-1 and reduce the harm caused by autophagy during MIRI. The A2aR/cAMP-PKA signal pathway and its regulated Bcl-2 were involved in the negative regulation of Beclin-1 in MIRI. Under other experimental conditions or parameters, this may need to discuss separately. For example, cAMP activates autophagy through an original pathway related to Beclin-1 in mesenchymal stem cells (73).

*In vivo* experiments shown that the area of myocardial infarction decreased and ventricular systolic dysfunction improved after A2aR activation. The contractile function of the left ventricle after myocardial infarction was closely relevant to the infarct size (74). Through cardiac ultrasound, we found that the activation of upstream A2aR and its downstream cAMP-PKA can enhance positive inotropic action. MIRI impaired the LVEF and LVFS, but A2aR activation effectively improved these two indicators of ventricular systolic function. In contrast, left ventricular systolic dysfunction was further deteriorative after using A2aR antagonist. Another study (75) mentioned that A2aR activation could improve myocardial systolic function far from the infarct area, but due to limited experimental equipment, we did not cover this index. The recovery of ventricular systolic function after MIRI depended on the elevation of cAMP and PKA induced by A2aR. The increased intracellular cAMP and PKA can trigger multiple cAMP-PKA-dependent ion channels, which heighten the maximum peak transient outward current and improve ventricular repolarization after ischemia (37, 38, 76). The research of Liang (77) considered that the positive inotropic effect of A2aR attributes to the activation of cAMP-dependent L-type calcium channels, which take a crucial place on CGS21680-induced increase of calcium influx. Kerfant et al. (78)found that regulation of cAMP can induce Ca^2+^ transients and absorb Ca^2+^ through SERCA2 (sarcoplasmic/endoplasmic reticulum Ca2+-ATPase 2) into the SER, thus enhancing the contractility of the myocardium. In conclusion, the cAMP-mediated Ca^2+^ influx contributes to the recovery of myocardial contractility.

Furthermore, MIRI caused ST-segment elevation and prolonged QT interval in Electrocardiogram. The interruption of blood flow and oxygen supply in ischemia and the changes in tissue perfusion during the reperfusion led to an imbalance in the inflow and outflow of intracellular and extracellular Ca^2+^, K^+^, H^+^, and Na^+^, resulting in changes in repolarization current (77, 79). This change of cell current in the IRI region prolonged the duration of the action potential, resulting in the prolonged QT and ST-segment elevation. Recent studies (62, 80) illustrated that AR activation during ischemic postprocessing had an antiarrhythmic effect, which was associated with action potential shortening. We also observed an improvement in QT interval prolongation following activation of A2aR with CGS21680. The cellular mechanisms of A2aR-mediated global ventricular repolarization and QT interval changes may refer to an augment in outward K^+^ current (37, 76, 81).

**Figure 8:**
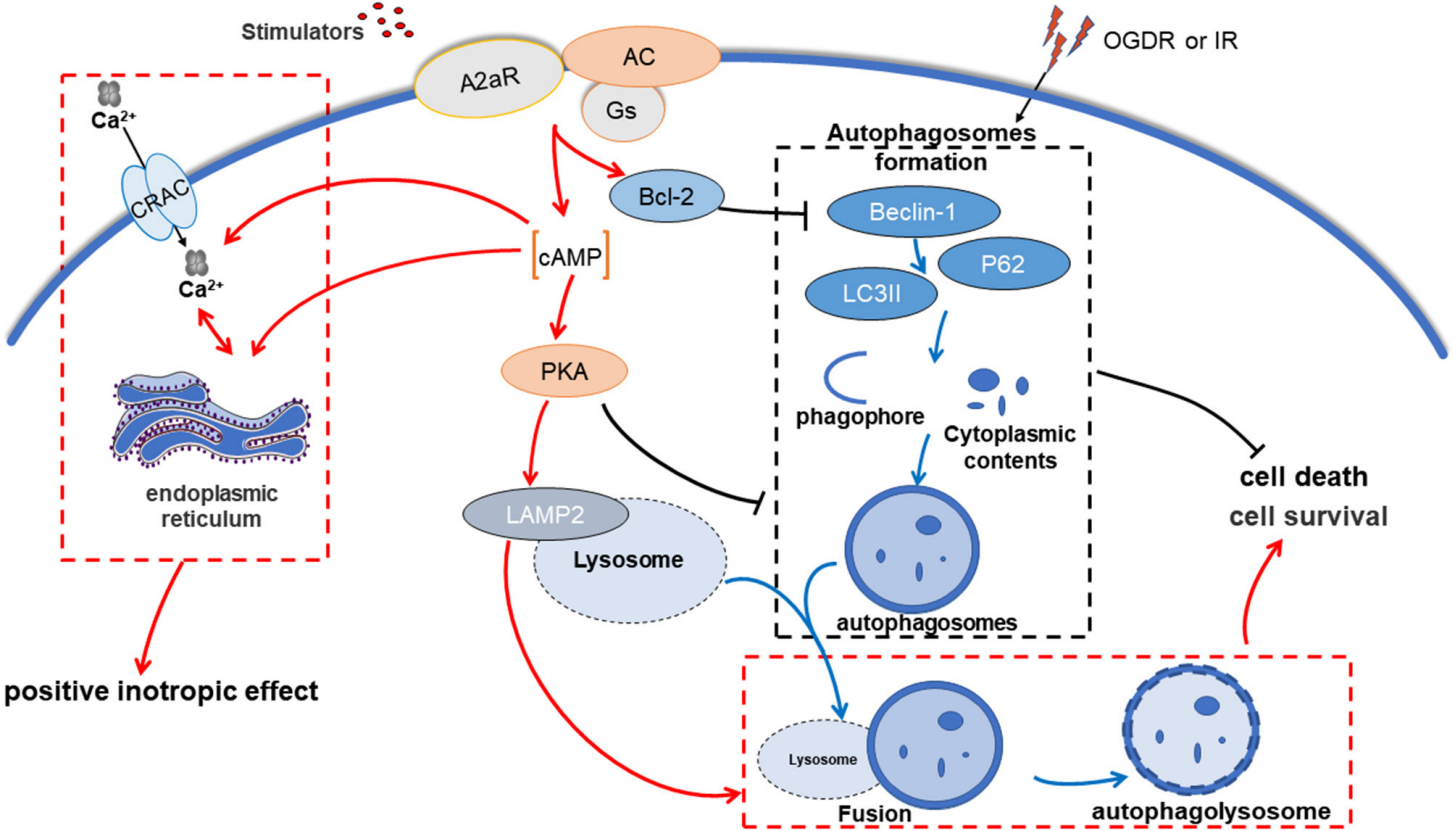
A2aR regulates autophagy and apoptosis to alleviate myocardial ischemia-reperfusion injury via cAMP-PKA. OGDR or IR damage induces intracellular accumulation of autophagosomes, which exacerbates cell death. In contrast, A2aR activation inhibits the generation of autophagosomes and promotes its cleaning process, allowing cells to survive.

In summary, A2aR activation before reperfusion can effectively inhibit apoptosis, reduce the formation of autophagosomes, and restore the impaired autophagy flux, thereby weakening ventricular dysfunction, improving QT interval prolongation, and reducing MIRI damage. This protective effect achieves by activating the cAMP-PKA pathway and Beclin-1-Bcl-2 complex related mechanisms (Figure 8).

## Data Availability Statement

The original contributions presented in the study are included in the article/Supplementary Material, further inquiries can be directed to the corresponding author/s.

## Ethics Statement

The animal study was reviewed and approved by the Animal Experiment Committee of Wuhan University (China, Approval No. WP2020–01108).

## Author Contributions

YXia, YXio, JZ, and HL: designed the study. YXia, MM, HZ, and JL: conducted experiments, collected, and analyzed data. YXia: wrote the manuscript. JK and YW: revised the manuscript. FH: supplemented the experiments. All authors read and final approval of manuscript.

## Funding

This work was supported by the National Natural Science Foundation of China (81871553).

## Conflict of Interest

The authors declare that the research was conducted in the absence of any commercial or financial relationships that could be construed as a potential conflict of interest.

## Publisher's Note

All claims expressed in this article are solely those of the authors and do not necessarily represent those of their affiliated organizations, or those of the publisher, the editors and the reviewers. Any product that may be evaluated in this article, or claim that may be made by its manufacturer, is not guaranteed or endorsed by the publisher.
