# Supplementary material for: Adenosine A2a Receptor Regulates Autophagy Flux and Apoptosis to Alleviate Ischemia-Reperfusion Injury via the cAMP/PKA Signaling Pathway
Source: Front Cardiovasc Med. 2022 Apr 29;9:755619. doi: 10.3389/fcvm.2022.755619 (PMC9099415; doi:10.3389/fcvm.2022.755619)
Supplement: Supplementary file 1 [file Data_Sheet_1.docx]

**Supplementary Table 1: Commodities, and chemicals used in this experiment.**

| **Reagents** | **Company** | **Catalog Number** |
| --- | --- | --- |
| **CGS21680** | **Tocris Bioscience** | **1036** |
| **ZM241385** | **Tocris Bioscience** | **1063** |
| **Dibutyryl-cAMP sodium salt** | **MedChemExpress** | **HY-B0764** |
| **H89** | **MedChemExpress** | **HY-15979** |
| **3-Methyladenine** | **MedChemExpress** | **HY-19312** |
| **Rapamycin** | **MedChemExpress** | **HY-10219** |
| **Trypsin** | **Yeasen** | **40101ES25** |
| **Collagenase type II** | **BioFroxx** | **9001-12-1** |
| **5-Bromo-2′-deoxyuridine** | **Yeasen** | **40204ES60** |
| **DMEM/F-12** | **Gibco** | **11320033** |
| **Advanced DMEM/F-12 Flex Media** | **Gibco** | **A2494301** |
| **Fetal bovine serum** | **Gibco** | **10100147** |
| **Penicillin-streptomycin solution** | **Beyotime Biotechnology** | **C0222** |
| **Evans Blue** | **Solarbio** | **E8010** |
| **TTC** | **Solarbio** | **T8170** |
| **Complete protease inhibitor** | **Roch** | **04693116001** |
| **Phosphatase inhibitor** | **Roch** | **04906837001** |
| **BCA Protein Assay Kit** | **CoWin Bio.** | **CW0014** |
| **Rat CK-MB Elisa Kit** | **Elabscience** | **E-EL-R1327c** |
| **Rat D-LDH Elisa Kit** | **Elabscience** | **E-EL-R0338c** |
| **Rat cTn-I Elisa Kit** | **Elabscience** | **E-EL-R1253c** |
| **Cell Counting Kit-8 assays kit** | **Meilun Biotechnology** | **MA0218** |
| **Ad-mCherry-GFP-LC3B** | **Beyotime Biotechnology** | **C3011** |

**Supplementary Table 2: Antibodies used in this study.**

| **Protein Name** | **Company** | **Catalog Number** |
| --- | --- | --- |
| **Mouse monoclonal anti-Bcl-2** | **Proteintech** | **60178-1-Ig** |
| **Mouse monoclonal anti-Bax** | **Proteintech** | **60267-1-Ig** |
| **Rabbit polyclonal anti-A2ar** | **Abcam** | **ab3461** |
| **Rabbit monoclonal anti-cAMP** | **Abcam** | **ab76238** |
| **Rabbit monoclonal anti-P62** | **Cell Signaling Technology** | **#23214** |
| **Rabbit polyclonal anti-LC3A/B** | **Cell Signaling Technology** | **#4108** |
| **Rabbit monoclonal anti-Beclin-1** | **Cell Signaling Technology** | **#3495** |
| **Rabbit polyclonal anti-pPKA** | **Cell Signaling Technology** | **#4781** |
| **Rabbit polyclonal anti-PKA** | **Cell Signaling Technology** | **#4782** |
| **Rabbit polyclonal anti-GAPDH** | **Primacy Biotechnology** | **PMK053S** |
| **Mouse polyclonal anti-β-Tublin** | **Primacy Biotechnology** | **PMK088S** |
| **TNNI3 Rabbit pAb** | **Abclonal** | **A6995** |
| **FITC goat anti-rabbit IgG** | **Abclonal** | **AS011** |
